# Research on charge induction law and application of coal samples with different fissures

**DOI:** 10.1038/s41598-023-42100-6

**Published:** 2023-09-20

**Authors:** Gang Wang, Siqi Gao, Aiwen Wang, Lianpeng Dai, Tianwei Shi, Zengjun Xu

**Affiliations:** 1 School of Environmental Science, Liaoning University, Shenyang, 110036 China; 2 Institute of Disaster Rock Mechanics, Liaoning University, Shenyang, 110036 China

**Keywords:** Natural hazards, Solid Earth sciences, Energy science and technology, Engineering, Physics

## Abstract

Indoor testing are performed to explore the charge induction law during the uniaxial compression fracture process of coal samples, and the charge time and frequency domain signals of coal samples with different primary fissures are analyzed in the paper. On-site monitoring of charge in different fissures distribution areas of underground coal tunnels, and the charge signals of different drillingdepths in coal seams are analyzed. The results show that the uniaxial compressive strength and elastic modulus of multi-fissured coal samples are less than those of less fissured coal samples, and the Poisson’s ratio is greater than those of less fissured coal samples. The charge induction signal intensity during the fracture process of multi-fissured coal samples is relatively low, but it is concentrated at the low frequency of 0–50 Hz in the compacting elasticity stage. The charge signal intensity during the fracture process of coal samples with less fissure is relatively high, and the charge frequency during the reinforcement damage stage is concentrated at a low frequency of 0–50 Hz. Therefore, the sudden appearance of low-frequency charge signals is more suitable as effective precursor information for the instability and failure of less fissured coal bodies. The average charge intensity is small in the multi-fissured area with a drilling depth of 1–4 m in the coal seam, and the average charge intensity of the coal body with less fissures is larger in the 5–12 m region. The on-site charge monitoring results have good consistency with the indoor test results. This study has guiding significance in setting up a charge monitoring warning index of instability failure in different coal body fissures regions.

## Introduction

The frequency and intensity of rock burst disasters has increased significantly with the annual increase in coal mining depth, which severely threatens safe and efficient mine production^1–5^. Therefore, achieving effective coal rock destabilization damage monitoring to ensure coal resources can be mined safely and efficiently has become an important scientific problem. The charge generated as a coal rock body is deformed and damaged due to the piezoelectric effect, frictional effect, and microrupture lead to tip charge separation, among others. The generated charge can be extracted and analyzed using a highly sensitive charge device^6–10^. The charge signal generated by the coal rock fracture is closely related to the coal rock dynamic process and contains extensive information on the mechanical process of coal rock deformation and rupture. The charge induction information can comprehensively convey the damage characteristics of each stage during rock burst, thus forming a novel rock burst monitoring method, namely charge induction monitoring, which is characterized by noncontact, continuity, and strong anti-interference.

Scholars have conducted numerous studies on charge-sensing information associated with coal rock deformation rupture. Nitson studied piezoelectric effects in rocks and found that when rocks containing quartz and other hard piezoelectric materials were fractured, charge-sensing signals were generated in the radio frequency band^11^. Kuksenko et al. measured the generation of induced charges with an electrostatic meter during the loading of marble and found that the amount of induced charges increased sharply when the rock was suddenly unloaded and before gradually decaying^12,13^. Triantis et al. observed a linear relationship between charge signal and deformation after applying uniform velocity stress to the rock under pressure stimulated currents (PSC)^14–16^. Vallianatos et al. presented an MCD model for generating electric current in rocks under stress, which was influenced by the motion of charge bearing dislocations. Within the MCD model, the relationship between current density and strain rate was first demonstrated and used as a prominent mechanism for producing electric earthquake precursors^17–19^. Varotsos et al. found that low-frequency, transient pulsed electrical signals were generated before an earthquake, and the signal duration was long^20^. Pan et al. investigated the charge induction signal of coal rock mass under uniaxial compression, triaxial compression, and tension conditions^21–23^. Zhao et al. conducted a simulated experimental study on the charge induction law of the fault slip process^24,25^. He et al. studied the charge induction characteristics of coal rocks with different degrees of metamorphism^26^. Wang et al. investigated the characteristics of charge signal evolution under mining damage in deep coal seams^27^. Lv et al. investigated the correspondence between the charge signal and energy accumulation and release of the coal body during coal body rupture^28^. Ding et al. studied the effect of water on the physical and mechanical properties and charge induction signal of coal bodies^29^. Wang et al. investigated the relationship between stress and charge during coal body rupture under uniaxial compression^30,31^.

In summary, scholars have conducted numerous studies on charge signal patterns of coal rock materials under external load actions, thus promoting the use of charge induction methods for predicting the coal rock dynamic hazards, such as rock burst. However, research on the charge induction law from the perspective of the fracture properties of the coal body is lacking. In addition, the coal seam contains different degrees of primary fractures due to deposition and crustal movement during formation processes, and the fracture assignment in the coal body determines its physical and mechanical properties, which in turn affect the damage characteristics of the coal body under load and instability^32,33^. Therefore, this paper aims to investigate the mechanical properties during uniaxial compression rupture and the charge time–frequency signal response law of coal body materials with primary fissures of varying degrees. Charge field monitoring in different fissure distribution areas and depth areas in coal mine tunnels is investigated to improve the charge sensing technology for monitoring and early warning of coal-rock dynamic disasters, such as rock burst.

## Materials and methods

### Specimen preparation

**Figure 1:**
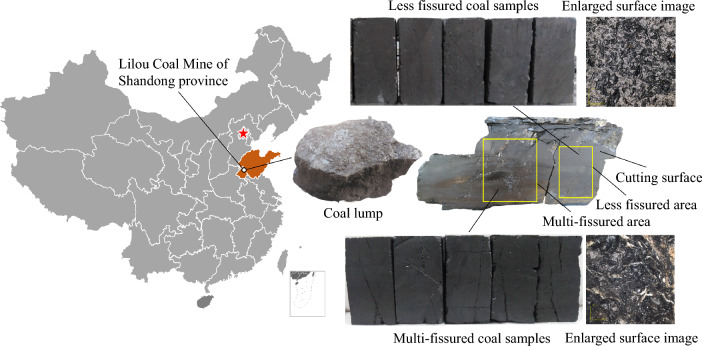
Coal samples with different primary fissures.

The coal samples were retrieved from a Li Lou coal mine in Shandong Province, China. The large coal blocks were cut open, revealing that they were neither intact nor homogenous but contained numerous cracks of different sizes. Two types of coal samples from areas with few cracks and areas with multiple cracks in coal blocks were prepared. The specimens were rectangular standard specimens of 50 mm × 50 mm × 100 mm length × width × height, as shown in Fig. 1. The surfaces of the two coal samples were observed under magnification using a microscope, which revealed that the surface material form of the Coal samples from less fissured areas were more uniform, and the pore structure was closely observed. The surface material form of the the Coal samples from multi-fissured areas were not uniform; rather, it contained numerous fissures and pore structures. Therefore, the two specimens differed in their nodal fissures.

The wave velocities of the two coal samples were measured using a nonmetallic ultrasonic detection analyzer, and the results are listed in Table 1. The acoustic wave velocity range of coal samples in the less fissured area is 1952–2079 m/s, while the acoustic wave velocity range of coal samples in the multi-fissured area is 1728–1871 m/s.

**Table 1:** Results of wave velocity measurement of coal samples.

Specimen type	Specimen number	Wave speed/(m/s)	Average wave speed/(m/s)
Less fissured coal samples	L-1	1952	1890
	L-2	1974	
	L-3	2079	
Multi-fissured coal samples	M-1	1734	
	M-2	1871	
	M-3	1728	

Noticeably, the less fissured coal samples had better interparticle cementation and higher homogeneity, and the acoustic wave propagated faster inside it. In contrast, the coal sample with many fissures had lower homogeneity, and the propagation of the acoustic wave in its interior was slower. Therefore, prominent differences in the internal structure of the two coal samples exist.This article defines coal samples with wave velocities exceeding 1890 m/s as less fissured coal samples, and coal samples with wave velocities less than 1890 m/s as multi-fissured coal samples.

### Experiment system

**Figure 2:**
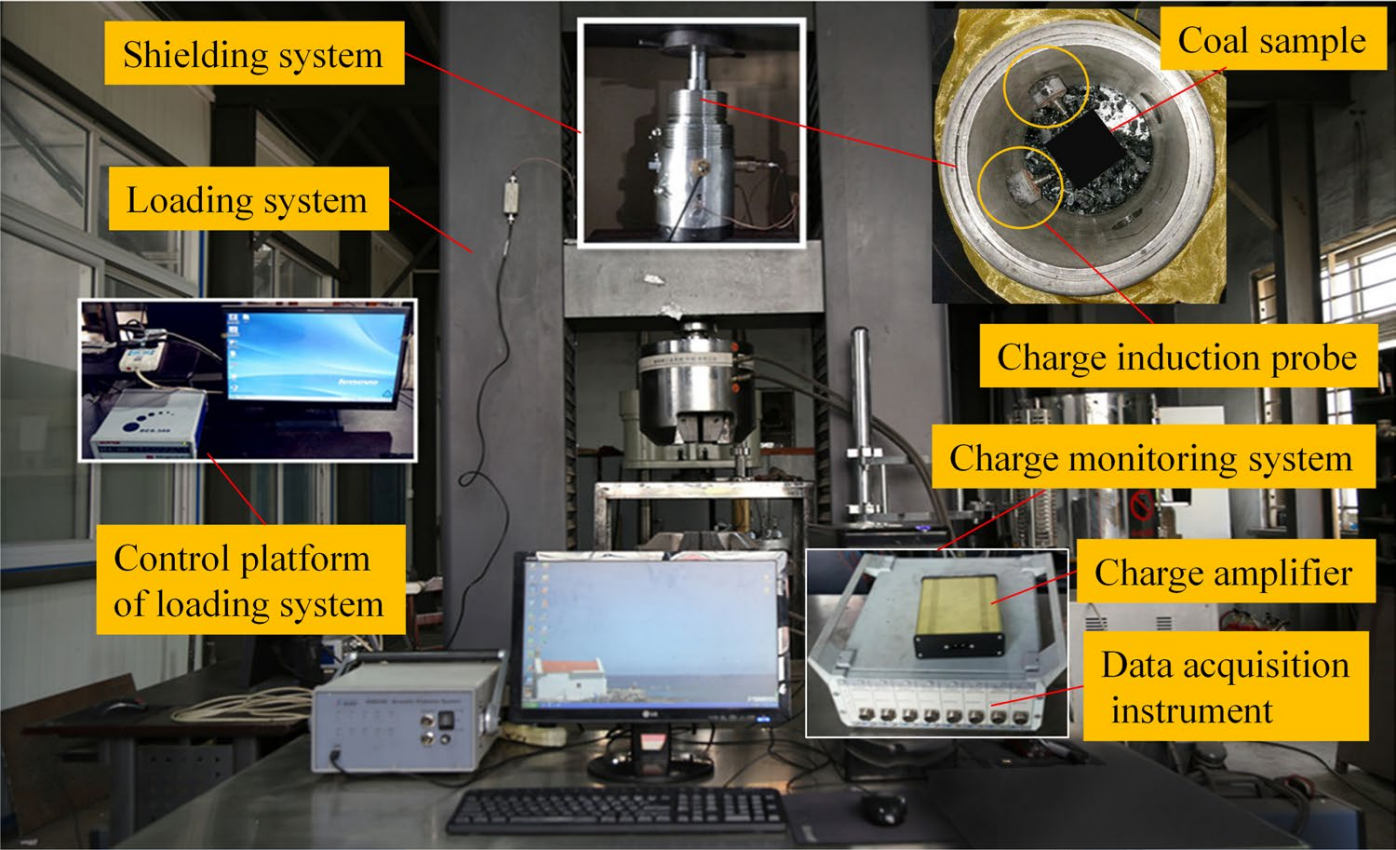
Monitoring test of induced charge in the process of coal failure.

As shown in Fig. 2, the experiment system includes a loading, shielding, and charge signal monitoring system.

A DCS-300 digital hydraulic servo test machine was used in the uniaxial compression test, and load and displacement data acquisition was achieved using automatic measurement devices. The machine has a compression capacity of 600 kN. The shielding system was a self-developed multifunctional cylindrical shielded steel cylinder with a diameter of 20 cm and a height of 30 cm. Moreover, insulating paper was used to insulate the bearing plate and coal samples. The charge signal monitoring system consisted of a noncontact charge induction probe, charge amplifier, and data acquisition instrument. The charge amplifier was ICA101, which was made in Hunan Province, China: its length, width, and height are 15 cm, 10 cm, and 3 cm. A sketch of the charge amplifier is shown in Fig. 2; the sensor frequency was 1–1000 Hz, and the sensitivity peak was > 75 dB^30^.

### Experiment principle and method

**Figure 3:**
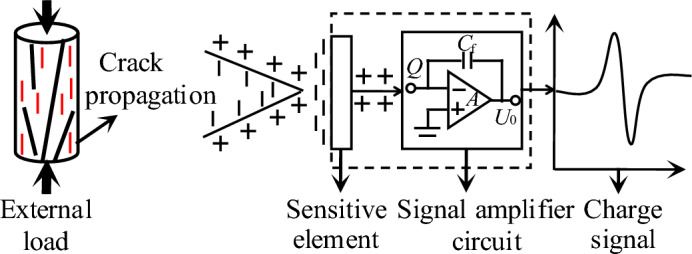
Coal–rock charge induction diagram.

As shown in Fig. 3, the coal mass generates free charge due to internal crack propagation and friction under external load. When charged particles on the crack surface pass through the sensitive element of the charge induction probe, the surface of sensitive element produces equivalent heterogeneous induced charges under the action of charge induction. When the charged particles on the crack surface are far from the sensitive element of the charge induction probe, the internal charge returns to an equilibrium state. The charge amplifier can convert and amplify the induced weak charge signal into a voltage signal through a certain proportional relationship. The voltage signal can then be collected using the data acquisition instrument. Thus, the deformation and failure information of the coal and rock mass can be obtained from the final signals^30^.

Displacement control under uniaxial compression load was adopted, and the displacement rate was 0.02 mm/s. The charge signal sampling frequency was set to 2500 Hz. The charge induction probe was placed on both sides of the sample, 5–10 mm away from the surface of sample, as shown in Fig. 2. The temperature and humidity were 26.5 °C and 37%, respectively.

### Signal processing

Due to the influence of the external environment, some interference signals are inevitably interspersed during the charge monitoring process, and their existence negatively affects the identification and extraction of effective signals of coal instability rupture. Accordingly, the rejection of noise interference signals is key to accurately identifying and analyzing the charge signals of coal instability rupture precursors. Therefore, this study adopts the Fourier transform method to analyze the noise and coal sample rupture signals in the frequency domain, explore the spectral distribution law of the two signals, and provide a basis for rejecting the noise signal.

Fourier transform is a method for converting the signal from time domain to the frequency domain. After the Fourier transform, the time domain signal can be decomposed into several sinusoidal components with different frequencies, amplitudes, and phases. Thus, the composition of the time domain signal sinusoid can be analyzed, and its distribution pattern in the frequency domain identified. Let X_(k)_ be the discrete Fourier transform of the charge signal x(n) with N sampling points, where k = 1, 2, 3, …, N − 1, then

1 $$ X\left( k \right) = \sum\limits_{n = 0}^{N - 1} {x\left( n \right)} \;{\text{e}}^{{ - {\text{j}}\frac{2\pi }{N}nk}} $$

### Experimental steps^30^

1. Install all test equipment and ensure their operation.
2. Place a coal sample into the shield cylinder and pad the insulation paper between the coal sample and pressure head to prevent charge overflow. Debug equipment and set parameters.
3. Start the loading system first and then the charge monitoring system.
4. Store the test result. Photograph coal sample destruction and process the data.

## Results and analysis

### Deformation damage and mechanical properties of coal samples

**Figure 4:**
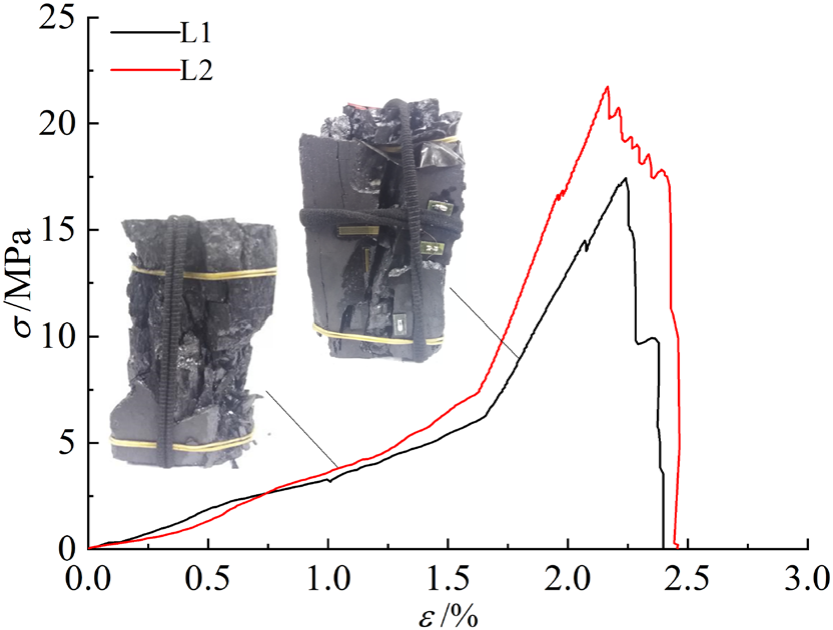
Micro-stress–strain curve of less fissured coal sample.

**Figure 5:**
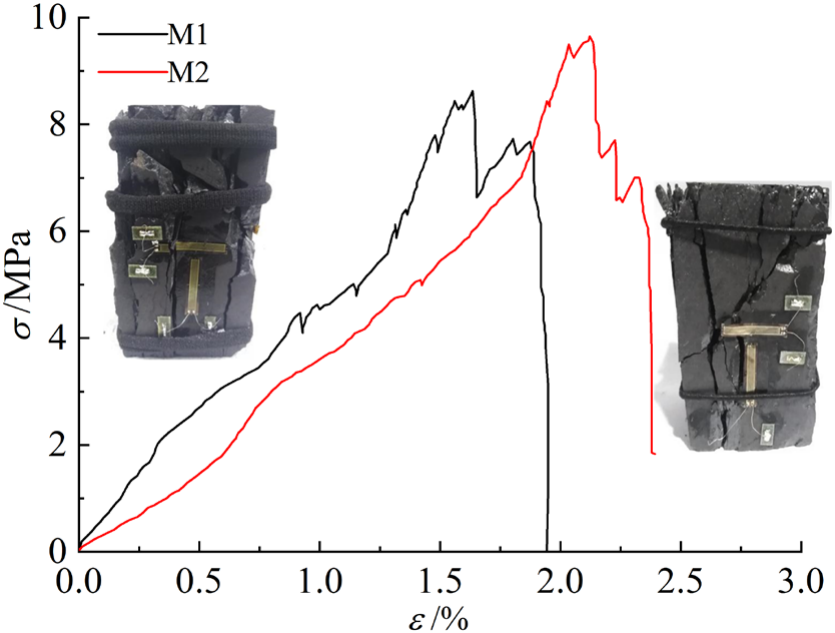
Micro-stress–strain curve of multi-fissured coal sample.

As shown in Fig. 4, the pre-peak stress–strain curve of the coal sample with less fissures is almost linearly elastic due to its better integrity, almost no stress drop occurs before the main rupture, and the post-peak stress–strain curve exhibits a vertical drop trend with prominent strain brittleness. The coal sample accumulated a large amount of elastic energy in the pre-stress peak phase, which was hardly consumed. When the stress reaches the ultimate strength, the load-bearing structural surface instantly destabilizes, accumulated elastic energy is rapidly released, coal sample undergoes single shear-type damage, large scale rupture occurs, sudden onset damage occurs, and the impact hazard is large. Figure 5 shows that, under low stress, multiple microruptures occurred in the multi-fissured coal sample due to its fissures, resulting in multiple adaptation of the stress–strain curve before the peak and partial release of elastic energy. After the stress peak was reached, the coal sample underwent progressive damage, and the stress–strain curve fell in a stepwise manner. The damage to the coal sample under load was not limited to one strip on the surface of the coal sample but multiple strips: multiple ruptures were conjugated, and the coal sample underwent conjugate shear-type damage.

**Figure 6:**
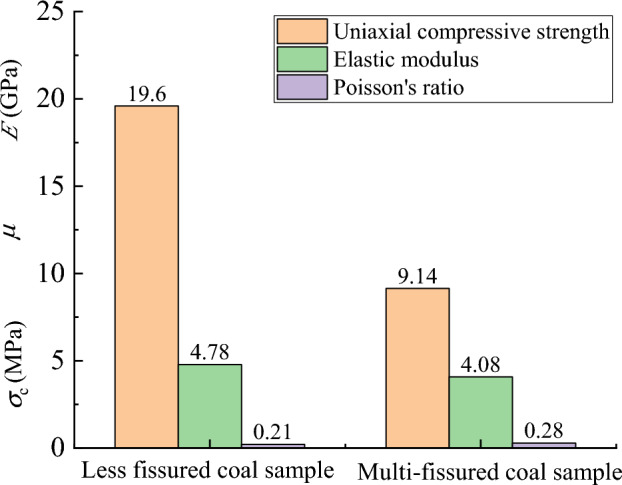
Comparison of mechanical properties of coal samples.

The mechanical properties of the two coal samples are listed in Table 2, and the data in the table are plotted in Fig. 6, which shows an average value of uniaxial compressive strength in the less fissured coal sample of 19.60 MPa, modulus of elasticity of 4.78 GPa, and a mean Poisson’s ratio of 0.21. In contrast, the mean uniaxial compressive strength of the multi-fissured coal samples was 9.14 MPa, which was 46.63% of that of the less fissured coal samples. Moreover, the mean elastic modulus was 4.08 GPa, which was 85.36% of that of the less fissured coal samples, and the mean Poisson’s ratio was 0.28, which was 1.33 times that of the less fissured coal samples.

**Table 2:** Mechanical parameters of different types of coal samples.

Coal sample type	Coal sample number	Uniaxial compressive strength/MPa	Average value/MPa	Elasticity modulus/GPa	Average value/GPa	Poisson’s ratio	Average value
Less fissure	L1	17.44	19.60	–	4.78	–	0.21
	L2	21.75		4.78		0.21	
Multi-fissure	M1	9.65	9.14	4.29	4.08	0.29	0.28
	M2	8.63		3.86		0.27

In summary, the deformation and damage characteristics and the mechanical properties of coal samples with few and multiple fissures differ markedly under external load mainly because the multiple fissure spaces inside the coal samples destroy their complete material structure, thus promoting the development and confluence of cracks until penetration damage occurs under smaller loads.

### Analysis of charge induction regularities

#### Charge time domain signal pattern analysis

**Figure 7:**
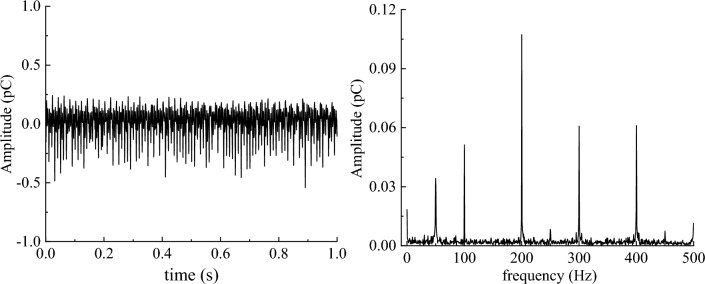
Time–frequency domain characteristics of coal sample noise signal.

**Figure 8:**
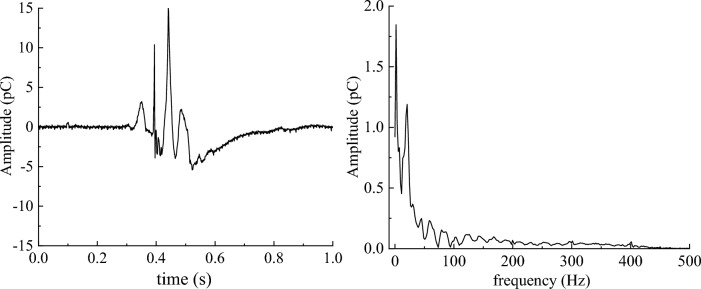
Time–frequency domain characteristics of the effective signal of coal samples.

The 1 s noise signal and 1 s coal sample rupture effective signal were subjected to a Fourier transform, and the time–frequency domain features are shown in Figs. 7 and 8, respectively.

As shown in Figs. 7 and 8, the frequencies of the noise signals were mainly distributed at 50 Hz, 100 Hz, 200 Hz, 300 Hz, and 400 Hz, and the main frequency was 200 Hz. The frequency of the effective signal of coal sample rupture was concentrated between 0 and 50 Hz, which is a low-frequency signal. Therefore, a low-pass filter can be used to filter the monitored original charge signal and filter out most high-frequency interference signals, namely those larger than 50 Hz.

**Figure 9:**
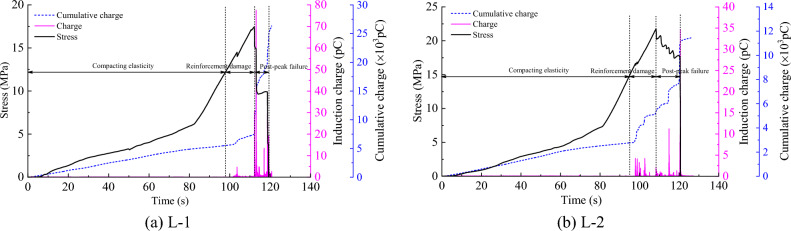
Stress-induced charge accumulation curve of less fissured coal sample with time.

**Figure 10:**
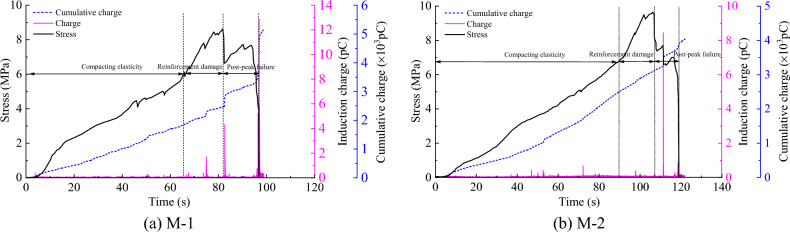
Stress-induced charge accumulation charge curve of multi-fissured coal samples with time.

The variation curves for the charge-sensing signal stress with time during uniaxial compression rupture of the two coal samples after denoising are shown in Figs. 9 and 10. According to the characteristics of the time–stress curve variation of the coal sample rupture, the deformation rupture process of coal samples can be divided into three stages: compacting elasticity (0–70%*σ*_c_), reinforcement damage (70%–100%*σ*_c_), and post-peak failure (100%*σ*_c_–0) stages. The charge time-domain signal pattern is analyzed below according to different failure stages.

Figure 9 shows that in the compacting elasticity stage of the less fissured coal sample, cumulative damage was small because the energy near the crack tip in the coal body was insufficient for microrupture expansion. Therefore, crack expansion was terminated, resulting in a very weak charge signal and a relatively smooth cumulative charge curve at this stage. With the gradual increase in stress, the coal sample entered the reinforcement damage stage. The sample accumulated enough energy at this stage, and the cracks inside the sample gradually formed and expanded. Accordingly, mutual friction between the internal particles of the sample was enhanced. The frequency and intensity of the charge signal increased; that is, the charge signal generated was consistently high, and the maximum signal magnitude was 4.2–4.7 pC. Notably, the cumulative charge curve was stepped up several times. As the stress increased, the coal sample enters the post-peak failure stage, the load-bearing structure surface was gradually damaged, and each stress drop corresponded to the generation of a charge signal, leading to a more intense charge signal due to a greater amount of energy being released after the peak of the coal sample. The maximum signal magnitude reached 34.7–77.6 pC, the cumulative charge curve continued to increase in multiple steps, and the step span increased.

Figure 10 shows that the multi-fissured coal sample underwent numerous instances of microfissure compression and microexpansion in the compacting elasticity stage due to the existence of multiple fissure spaces, resulting in charge signal generation due to friction and other effects. The maximum signal magnitude was between 0.3 and 0.7 pC, and the cumulative charge curve stabilized after several abrupt changes. With the gradual increase in stress, the coal sample entered the reinforcement damage stage, which was characterized by the convergence and penetration of numerous microruptures, and the accumulated damage increased, leading to a gradual increase in the number of charge signals. Moreover, the amplitude of the charge signals at this stage increased, and the maximum signal amplitude was between 0.4 and 1.7 pC; likewise, the accumulated charge curve rises in a stepwise manner. As the stress continued to increase, the coal sample entered the post-peak failure stage, and the coal sample was ruptured several times in the first two stages due to the existence of numerous fracture spaces, thus releasing more energy. Therefore, the frequency and intensity of charge signal generation decreased in the post-peak damage stage in the multi-fissured sample compared to in the less fissured coal sample. The maximum magnitude was between 8.4 and 12.8 pC, and the cumulative charge curve exhibited a small step increase.

In summary, charge signals were generated during the deformation and rupture of both coal samples, and the charge signals tended to gradually increase with the increase in load. Notably, the generation of charge signals and the sudden change in the amount of accumulated charge were in good agreement with the stress changes. The fractured coal samples exhibited marked charge signal generation in the compacting elasticity stage, but due to the presence of multiple fractures, the stress in the samples was lower. Thus, the intensity of the charge signal weakened during the entire rupture process than in the less fissured coal samples. However, under the same loading conditions, the stress level that the coal sample can withstand is related to its own properties. Therefore, the fundamental reason for the difference in charge law between multi-fissured and less fissured coal samples is the different characteristics of the their fissures. Based on experimental results, when the charge signal intensity is utilized for early warning of coal body destabilization damage, different warning indicators should be applied to different fracture areas.

#### Analysis of charge frequency domain signal pattern

**Figure 11:**
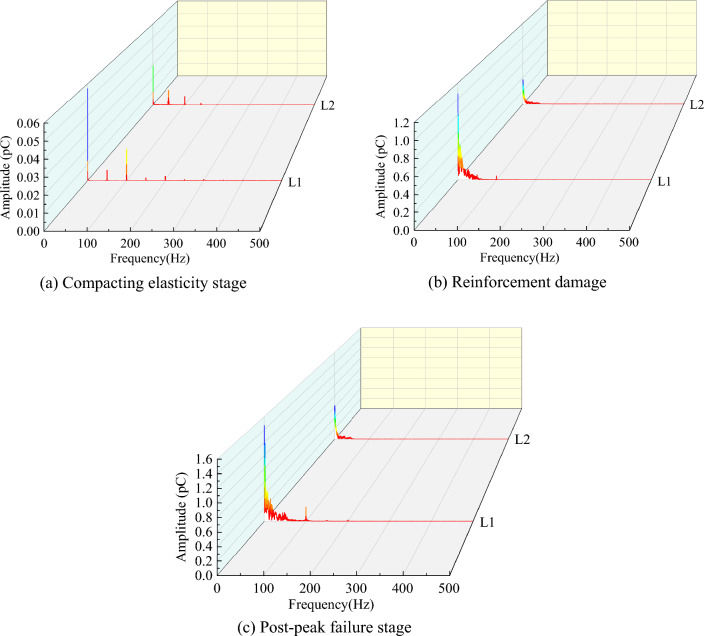
Frequency domain signals of charge at different fracture stages for the less fissured coal samples.

**Figure 12:**
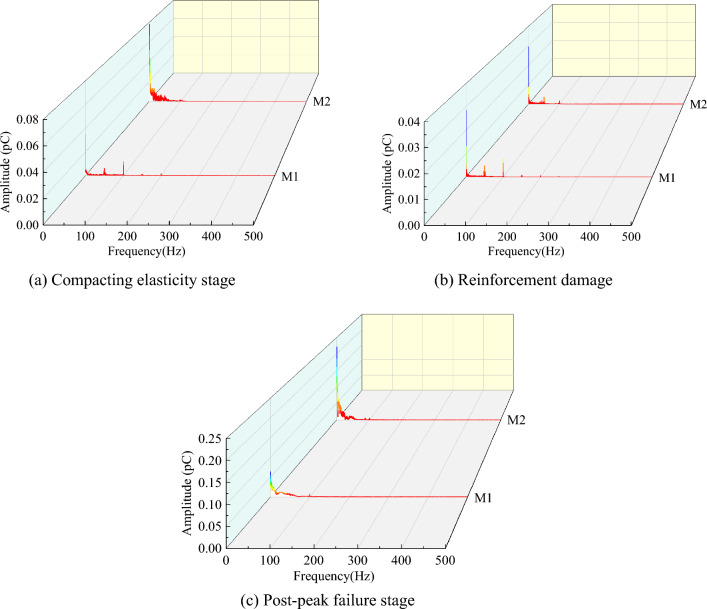
Frequency domain signals of charge at different fracture stages of multi-fissured coal samples.

A frequency domain analysis of the charge signal was performed for different fissures coal samples in the compacting elasticity, reinforcement damage and post-peak failure stages, as shown in Figs. 11 and 12, respectively.

As shown in Figs. 11 and 12, the charge frequencies of the compacting elasticity stage for the less fissured coal samples were mainly distributed in the range of 0–200 Hz. The charge frequencies of the multi-fissured coal samples were mainly distributed in the range of 0–100 Hz. However, these were concentrated in 0–50 Hz range, and the amplitude between 0 and 50 Hz was higher than that of the less fissured coal samples. This phenomenon is attributable to the presence of more microfractures inside the multi-fissured coal sample and the low crack initiation stress. Therefore, crack expansion occurs in the compression-density elastic stage with low stress, resulting in the generation of the effective low frequency signal. On the contrary, the less fissured coal sample appeared as almost a noise signal at this stage.

In the reinforcement damage stage, the charge frequencies of the less fissured coal samples were mainly concentrated in the range of 0–50 Hz. The charge frequencies of the multi-fissured coal samples were still mainly distributed in the range of 0–100 Hz. This is attributed to the stress accumulation in the less fissured coal sample occurring in the compression-dense elastic stage, whereas crack sprouting and expansion begin in the reinforced damage stage. Thus, the effective low-frequency signal is generated with high intensity. In contrast, the multi-fissured coal sample begins to crack and expansion and stress release occur due to the compression-dense elastic stage, resulting in further fusion and penetration of the fissures at this stage. Moreover, the effective charge signal was generated, but the charge intensity was weak; and the weak noise signal was entrained.

In the post-peak failure stage, the charge frequencies of both coal samples are concentrated in the range of 0–50 Hz, and the amplitudes are higher than those in the reinforcement damage stage. However, the amplitudes are significantly higher than those of the multi-fissured coal samples due to the large amount of energy released from the less fissured coal samples.

In summary, deformation and rupture in a multi-fissured coal body tends to be homogeneous due to the fissure space structure present, which leads to a charge frequency concentration in the low frequency range of 0–50 Hz in the compacting elasticity stage. Moreover, the charge frequency domain exhibits a similar pattern in the reinforcement damage stage and is concentrated at 0–50 Hz in the post-peak damage stage. On the contrary, the deformation and rupture process in the less fissured coal body exhibited marked abruptness due to its better integrity, with stress accumulation in the compacting elasticity stage, sudden stress release in the reinforcement damage stage, and charge frequency concentration in the low frequency range of 0–50 Hz. Therefore, when charge frequency is used for coal body destabilization rupture warning analysis, the sudden appearance of low-frequency signals is suitable as warning information for destabilization damage in coal bodies with few fissures. However, utilizing low-frequency signals in multi-fissured coal bodies may result in a transition warning phenomenon.

## Application practice

After the of coal seam roadway is excavated, the degree of rupture in the coal body near the coal wall of the roadway increases due to the stress perturbation effect, creating a multi-fissured area in the coal body. Thus, the rupture degree gradually decreases away from the roadway, resulting a less fissured area in the coal body. To investigate the charge signal pattern in different fracture areas of the underground coal body and verify the laboratory test results, a field coal seam roadway charge monitoring test was conducted at different drilling depths.

### Charge monitoring equipment

**Figure 13:**
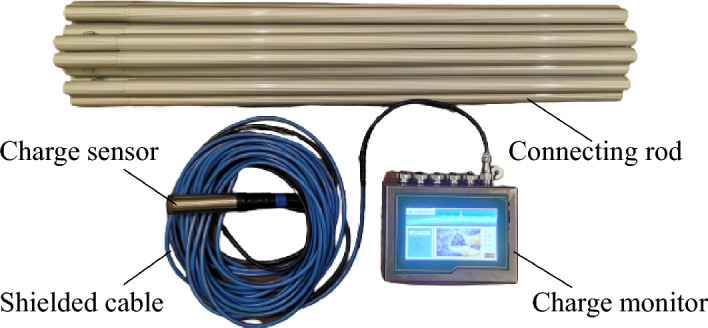
Portable coal and rock charge monitor.

An independently developed portable coal and rock charge monitor was used in the field test for charge monitoring, as shown in Fig. 13. The visualization, multichannel, and portable equipment consisted of a charge sensor with a shielded cable and a data acquisition instrument. It can display the change law of the charge signal and evaluate the fractured state of coal and rock in real time and had four monitoring channels, which can simultaneously monitor multiple underground measuring points. The sampling accuracy was 16 bits, and it was run continuously for more than 8 h. The sampling frequency was 1000 Hz, at which the maximum value was recorded. The measuring range was from − 75 to 75 pC. The operating temperature range was from − 20 to 60 °C. The length, width, and height of the equipment were 20 cm, 15 cm, and 10 cm, respectively, and it weighed 2 kg, making it easy to carry.

### Monitoring site and monitoring point layout

**Figure 14:**
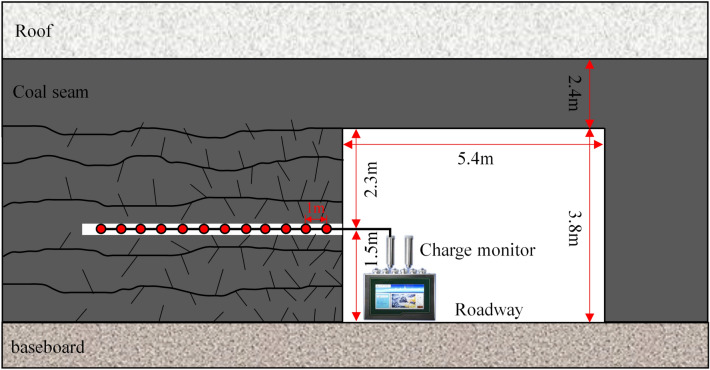
Schematic of the field charge monitoring program.

**Figure 15:**
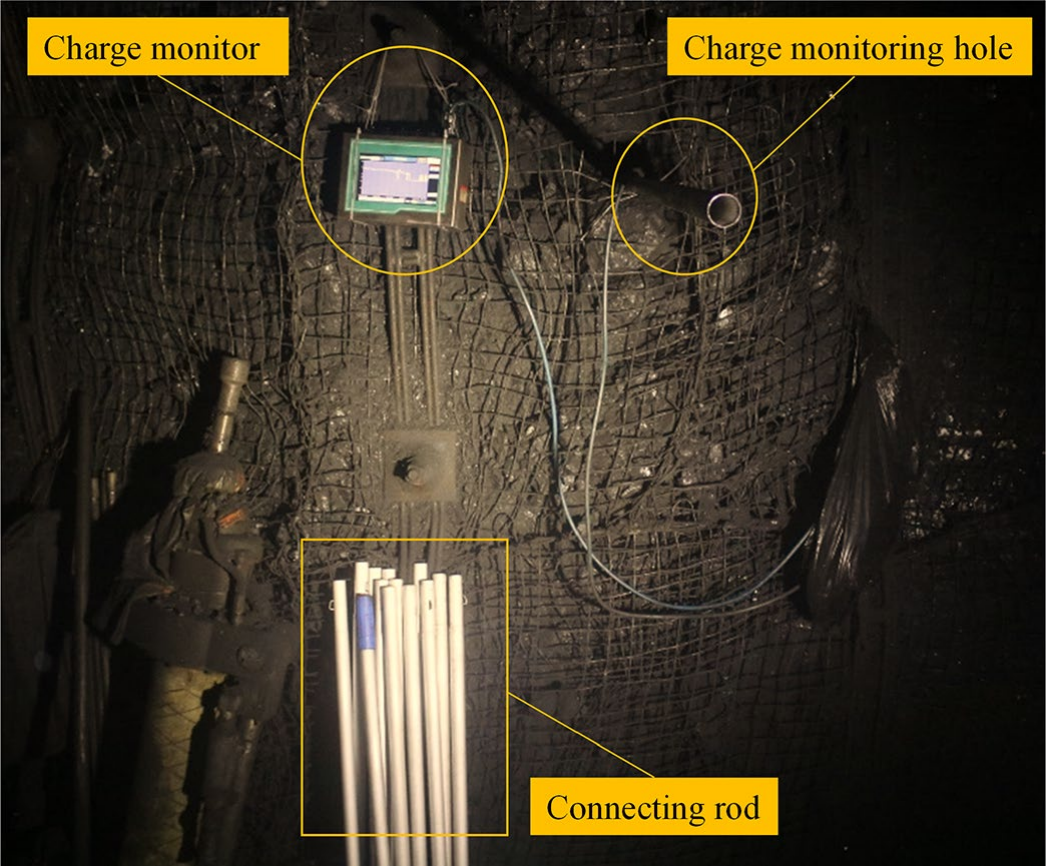
Field charge monitoring process diagram.

The charge monitoring site was located in a coal seam tunnel of a mine in China. The tunnel depth, coal seam thickness, tunnel height, and tunnel width were 600 m, 6.2 m, 3.8 m, and 5.4 m, respectively. The vertical coal wall was drilled into the coal seam 1.5 m from the bottom of the tunnel, and the hole depth was 15 m. After the stress was balanced, the charge sensing probe was inserted into the monitoring hole. Charge monitoring was performed every 1 m from the outside to the inside for a total of 12 m, and the monitoring time was 3–5 min. A schematic of the charge monitoring scheme is shown in Fig. 14, and the field monitoring process is shown in Fig. 15.

### Monitoring results and analysis

**Figure 16:**
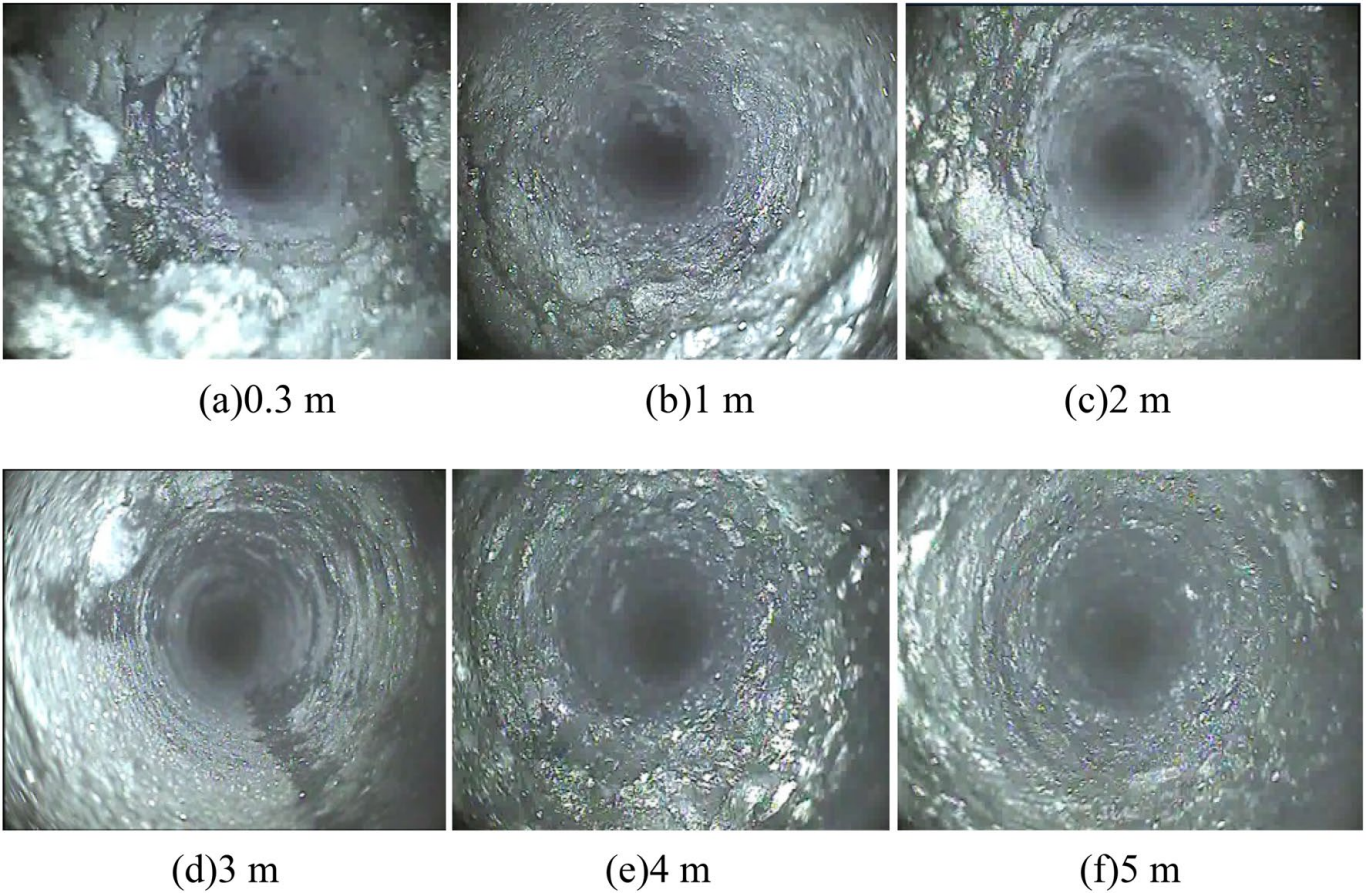
Peephole results for coal body at different drilling depths.

The peeping results for different borehole depths are shown in Fig. 16 (beyond a 5 m depth, coal dust in the borehole prevents further peeping). Noticeably, the coal body at the mouth of the hole had the greatest degree of rupture and the most fissures, which both gradually decreased as the depth gradually increased. When the borehole depth reached 5 m, the fissures significantly decreased. Therefore, the region between 0 and 5 m of the borehole can be regarded as multi-fissured and that beyond 5 m as less fissured. By monitoring the charge signal at different depths, the change law of the charge signal in different coal fracture areas can be explored.

**Figure 17:**
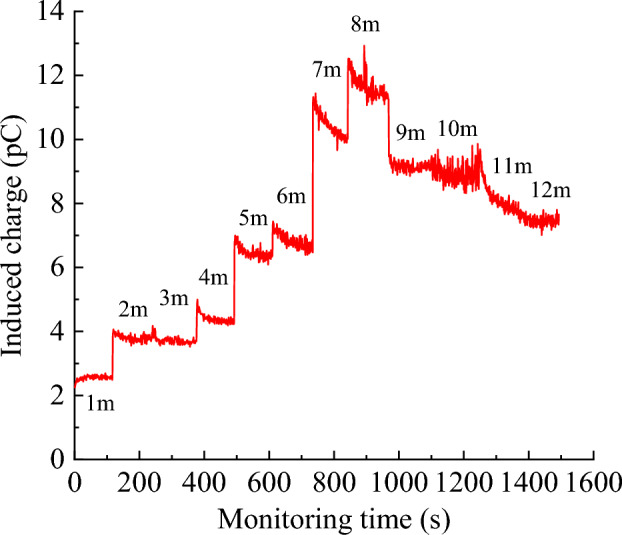
Real-time charge data graph.

**Figure 18:**
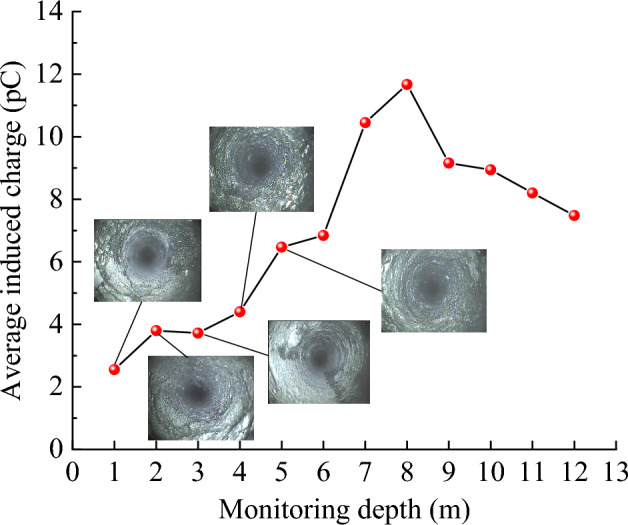
Monitoring depth versus charge change correspondence.

The real-time monitoring results of the charge signal intensity at different borehole depths are shown in Fig. 17, and the average charge intensity at different borehole depths was obtained after averaging the real-time charge intensity at each depth, as shown in Fig. 18. The coal fissure development was significant at a monitoring depth of 1 m, the charge induction signal was relatively smooth, and the average charge intensity was 2.55 pC. At a monitoring depth of 2, 3, and 4 m, the coal fissures decreased slightly with the increase in drilling depth; the charge signal intensity was enhanced; and the average charge intensities were 3.80 pC, 3.72 pC, and 4.40 pC, respectively. At a monitoring depth of 5 m, the fissures in the coal body significantly reduced; the charge induction signal was markedly enhanced; and the average charge intensity suddenly increased to 6.47 pC, which was 1.79 times the average charge intensity of 1–4 m. Coal fissures further reduced at monitoring depths between 5 and 12 m, and the charge signal was further enhanced, with the maximum average charge intensity being 11.67 pC. In summary, when the charge monitoring range was the multi-fissured area of coal body from 1 to 4 m, the stress level of the coal body was lower and the average charge intensity was weaker than when the charge monitoring range in the less fissured coal body was from 5 to 12 m. Therefore, the charge monitoring results for different coal fracture areas in the field agreed with the indoor test results. This law can serve as a guideline for setting early warning indicators of destabilization damage charge monitoring in different coal fracture areas in the field and applying charge sensing technology for detecting coal seam fracture areas.

## Discussion

Scholars have studied the self potential, charge induction, and electromagnetic radiation laws during uniaxial compression fracture of artificially pre cracked coal samples. They mainly analyzed the influence of changes in the inclination angle of prefabricated cracks on the electrical signals during the coal sample fracture process. The findings of their research can serve a guideline to monitor the destabilization damage of macrogeological tectonic coal rock bodies^34–36^.

In this paper, the charge induction law of the fracture process of different primary fissure coal samples is investigated, and the selected coal sample morphology is more in line with the actual geological conditions of the coal body. We not only conducted indoor experiments but also conducted on-site charge monitoring experiments, and have added charge frequency domain analysis in the resultes analysis section. The charge monitoring results in the field agree with the indoor test results. Therefore, when utilizing charge signal intensity for coal body destabilization damage prediction analysis, different warning indicators should be set for different fissure regions in the coal seam. Meanwhile, based on the experimental results, we conclude that when utilizing charge intensity for coal body destabilization damage prediction, different warning indicators should be set for different fissure regions. When utilizing charge frequency for coal body instability rupture prediction, the sudden appearance of low-frequency signals is more suitable as effective precursor information for instability damage in coal bodies with few fissures. Through discussion, the research results of this paper can improve the accuracy of charge induction technology for detecting coal seam fracture areas.

## Conclusions

1. Uniaxial shear damage generally occurs in less fissured coal samples, whereas conjugate shear damage occurs in multi-fissured coal samples. The compressive strength and elastic modulus of the multi-fissured coal samples are smaller than those of the less fissured coal samples, but the Poisson’s ratio is larger than that of the less fissured coal samples.
2. The charge signal during the fracture of multi-fissured coal samples is widely distributed, but the overall intensity of the charge signal is low. Meanwhile, the charge signal of less fissured coal samples starts to appear in the reinforced damage stage, and the overall intensity of charge signal is high. Different charge intensity warning indicators should be set for predicting instability in different coal fissure areas.
3. The low-frequency charge signal is generated in the compacting elasticity stage for multi-fissured coal samples, while less fractured coal samples generate during the reinforcement damage stage. The presence of a low-frequency charge signal is suitable as effective precursor information of destabilization damage in a less fissured coal body, but its application in a multi-fissured coal body may lead to over-warning.
4. As the depth of coal seam drilling increases, coal body fissuring gradually decreases, and the corresponding average charge signal intensity per meter gradually increases. The charge induction technology can be applied to the preliminary detection of coal seam fissure areas to guide coal seam geological structure exploration.

## Data Availability

The data used to support the findings of this study are available from the first author upon request. (Wang Gang: wg_0404@163.com).
